# Significant valvular heart disease in wild-type transthyretin amyloidosis: prevalence and impact on survival

**DOI:** 10.47487/apcyccv.v6i3.524

**Published:** 2025-09-24

**Authors:** Santiago Decotto, Pilar Domenech, Pedro Touzas, María Adela Aguirre, Elsa Nucifora, María Lourdes Posadas-Martínez, Rocío Blanco, Mariano Falconi, Rodolfo Pizarro, Diego Pérez de Arenaza

**Affiliations:** 1 Servicio de Cardiología, Hospital Italiano de Buenos Aires, Ciudad Autónoma de Buenos Aires, Argentina. Servicio de Cardiología Hospital Italiano de Buenos Aires Buenos Aires Argentina; 2 Servicio de Clínica Médica, Hospital Italiano de Buenos Aires, Ciudad Autónoma de Buenos Aires, Argentina. Universidad de Buenos Aires Servicio de Clínica Médica Hospital Italiano de Buenos Aires Buenos Aires Argentina; 3 Servicio de Hematología, Hospital Italiano de Buenos Aires, Ciudad Autónoma de Buenos Aires, Argentina. Servicio de Hematología Hospital Italiano de Buenos Aires Buenos Aires Argentina; 4 Instituto de Medicina Traslacional e Ingeniería Biomédica (IMTIB), Ciudad Autónoma de Buenos Aires, Argentina. Instituto de Medicina Traslacional e Ingeniería Biomédica (IMTIB) Buenos Aires Argentina

**Keywords:** Amyloidosis, Transthyretin, Valvular Heart Diseases, Tricuspid regurgitation, Amiloidosis, Transtiretina, Enfermedades de las Válvulas Cardíacas, Insuficiencia de la Válvula Tricúspide

## Abstract

**Objective.:**

There are limited data on the prevalence and prognostic impact of significant valvular heart disease in patients with wild-type transthyretin cardiac amyloidosis (ATTRwt). The aim of this study was to describe the prevalence of aortic stenosis (AS), mitral regurgitation (MR), and tricuspid regurgitation (TR) in this population and, secondarily, to evaluate their impact on survival.

**Methods.:**

This was a retrospective, single-center cohort study including patients diagnosed with ATTRwt between 2011 and 2024. We calculated the prevalence of each significant valvular heart disease, and Kaplan-Meier survival curves were generated to estimate two-year all-cause mortality, stratified according to the presence or absence of each significant valvular disease. Finally, a multivariable Cox regression was performed to assess their association with survival.

**Results.:**

We included 154 patients, with a mean age of 81 (±7) years; 85% (n=131) were male. Significant TR was the most prevalent valvular disease (27%, n=41), followed by significant MR (20%, n=30) and AS (13%, n=20). Patients with significant TR had a higher incidence of all-cause mortality compared to those without significant TR (41% vs. 16%, log-rank test, p=0.0007), whereas no differences were observed between patients with and without significant AS or MR. In multivariable Cox models, significant TR was independently associated with higher mortality, regardless of age, the presence of other valvular diseases, and NT-proBNP levels.

**Conclusions.:**

Significant TR was the most prevalent valvular disease in patients with ATTRwt and was associated with lower survival in this population.

## RESUMEN

**Objetivo.:**

Existen pocos datos sobre la prevalencia de valvulopatías significativas en pacientes con amiloidosis cardíaca por transtiretina *wild type* (ATTRwt). El objetivo de este estudio fue describir la prevalencia de estenosis aórtica (EA), insuficiencia mitral (IM) e insuficiencia tricuspídea (IT) en esta población y, de forma secundaria, evaluar su impacto en la sobrevida.

**Materiales y métodos.:**

Estudio de cohorte retrospectivo, unicéntrico, que incluyó pacientes con diagnóstico de ATTRwt entre 2011 y 2024. Se calculó la prevalencia de cada valvulopatía significativa y se elaboraron curvas de Kaplan-Meier para estimar la incidencia de muerte por cualquier causa a dos años de seguimiento, estratificada por la presencia o ausencia de cada una de las valvulopatías. Finalmente, se realizó un análisis multivariado de Cox para evaluar su relación con la sobrevida.

**Resultados.:**

Se incluyeron 154 pacientes; la edad media fue de 81 (±7) años y el 85% (n=131) fueron hombres. La IT significativa fue la valvulopatía más prevalente (27%, n=41), seguida por la IM (20%, n=30) y la EA (13%, n=20). Los pacientes con IT significativa presentaron una mayor incidencia de mortalidad por cualquier causa comparados con los pacientes sin IT significativa (41% vs. 16%, Log-Rank p=0,0007), y no se observaron diferencias entre los pacientes con y sin EA e IM significativas. En los modelos multivariados, la IT significativa se asoció con mayor mortalidad independientemente de la edad, de la presencia de otras valvulopatías y de los niveles de NT-proBNP.

**Conclusiones.:**

La IT significativa fue la valvulopatía más prevalente en pacientes con ATTRwt y se asoció a una menor sobrevida en esta población.

## Introduction

Wild-type transthyretin amyloid cardiomyopathy (ATTRwt-CM) has become an increasingly recognized cause of heart failure (HF), ^1^ particularly in patients with preserved ejection fraction. ^2^^-^^4^ In recent years, its diagnosis has become more frequent, largely owing to the introduction of bone scintigraphy with bisphosphonates as a non-invasive detection method and the development of disease-modifying therapies. ^5^^,^^6^

Several prognostic staging systems for ATTRwt-CM have been developed in recent years, based on circulating biomarkers such as NT-proBNP, high-sensitivity troponin, and glomerular filtration rate. ^7^^,^^8^ However, other echocardiographic structural findings, such as the presence of significant valvular heart disease, have not been incorporated into these tools, despite their potential clinical relevance.

Valvular involvement in patients with ATTRwt-CM has been scarcely described. Although abundant evidence exists on the coexistence of aortic stenosis (AS) and ATTRwt-CM, ^9^ particularly in elderly patients with severe AS evaluated for valve replacement, ^10^^-^^12^ little is known about the presence and impact of other significant valvular diseases in this population, such as mitral regurgitation (MR) or tricuspid regurgitation (TR). These valvular abnormalities may represent either direct manifestations of amyloid infiltration or hemodynamic consequences of ventricular dysfunction or cardiac chamber remodeling.

Given the rising prevalence of ATTRwt-CM and the potential clinical impact of concomitant valvular disease, it is of particular interest to characterize the presence of significant valvular abnormalities in this population and their association with prognosis. The aim of this study was to describe the prevalence of significant AS, MR, and TR in patients with ATTRwt-CM and to assess their relationship with mid-term survival.

## Materials and methods

### Study design

We conducted a retrospective, single-center cohort study using data from the Institutional Amyloidosis Registry (RIA) of Hospital Italiano de Buenos Aires (NCT01347047), which includes consecutive incident cases from 2010 to 2024. This registry contains clinical, biochemical, imaging, and follow-up information from patients diagnosed with amyloidosis.

### Study population

We included patients with a confirmed diagnosis of ATTRwt-CM recorded in the RIA between 2011 and 2024. Diagnosis was established by:

- Positive bone scintigraphy with bisphosphonates (uptake grade ≥2 on the Perugini visual scale) and confirmatory myocardial uptake on SPECT-CT, in the absence of hematological findings suggestive of light-chain amyloidosis.
- In cases diagnosed before 2017 (when bone scintigraphy with pyrophosphate was introduced in our institution), the diagnosis was accepted if a positive endomyocardial or extracardiac biopsy with Congo red staining was present, together with typical findings on cardiac magnetic resonance imaging and negative hematological studies.

Patients with other forms of amyloidosis, including hereditary amyloidosis (ATTRv), and those diagnosed before 2011 (due to limited availability of echocardiographic data in the institutional electronic health record), were excluded.

### Variables

We collected demographic information (sex, age at diagnosis), associated comorbidities (hypertension, dyslipidemia, diabetes mellitus, etc.), laboratory data (serum creatinine, NT-proBNP, and high-sensitivity troponin), and routine echocardiographic variables. Significant valvular heart disease was defined as moderate or severe, according to current echocardiography practice guidelines ^13^^,^^14^.

Data used for this study were obtained from the RIA and the institutional electronic health record.

### Ethical aspects

The study protocol follows the ethical guidelines of the 1975 Declaration of Helsinki. The RIA was approved by the institutional ethics committee (protocol number 1975), and all participants provided informed consent before enrollment in the registry. 

### Data analysis

Continuous variables were expressed as mean ± standard deviation (SD) or as median and interquartile range (IQR), as appropriate. Categorical variables were expressed as percentages. Comparisons between patients with and without each of the three valvular heart diseases were performed using the chi-square test for categorical variable and Student’s t-test or the Mann-Whitney U test for continuous variables, according to parametric or non-parametric distribution, respectively.

Kaplan-Meier curves were generated to estimate all-cause mortality at 2 years after ATTRwt-CM diagnosis, stratified by the presence or absence of each valvular disease, as well as by the number of valvular diseases (0, 1, or 2). Multivariable Cox models were then constructed, including the three valvular diseases and other clinically relevant variables, to evaluate their independent association with survival. A two-sided p-value <0.05 was considered statistically significant. All analyses were performed using STATA version 13.1 (Stata Corp LP, College Station, TX).

## Results

### General characteristics of the cohort

A total of 154 patients were included. The mean age was 81 (SD: 7) years, and 85% (n=131) were men. Regarding comorbidities, 81% (n=126) had hypertension, 50% (n=78) had dyslipidemia, 22% (n=34) had diabetes mellitus, and 19% (n=29) had a history of coronary artery disease. More than half of the patients (54%, n=84) had permanent or paroxysmal atrial fibrillation, and 66% (n=102) had signs or symptoms of heart failure at the time of diagnosis, with 38% (n=59) in New York Heart Association (NYHA) class III/IV. Median NT-proBNP was 2976 (IQR: 1345-5494) pg/mL, and median high-sensitivity troponin was 55 (IQR: 37-88) pg/mL.

With respect to echocardiographic characteristics, the median left ventricular ejection fraction (LVEF) was 53% (IQR: 45-60), and the mean septal thickness was 17 (SD: 4) mm. Twenty-nine percent (n=45) presented with a single isolated significant valvular disease, 14% (n=22) had two valvular diseases, and only two patients presented with all three simultaneously. Significant TR was the most prevalent valvular disease (27%, n=41), followed by MR (20%, n=30) and AS (13%, n=20). Regarding etiology, AS was degenerative in all patients, and significant MR and TR were functional (ventricular or atrial) in all cases. Only 18% (n=28) of patients received disease-modifying therapy (stabilizers in all cases), with no differences between those with and without each significant valvular disease. As for valvular interventions, only six patients with significant AS underwent an aortic valve procedure (three transcatheter aortic valve replacements, one conventional replacement with a rapid-deployment prosthesis, and two balloon valvuloplasties without definitive treatment). No patients underwent mitral or tricuspid valve interventions.

### Characteristics of patients with and without significant valvular disease

When comparing clinical and imaging characteristics of patients with or without significant valvular disease, no statistically significant differences were observed between those with and without significant AS **(**Table 1**)**.

**Table 1:** Characteristics of patients with and without significant aortic stenosis at the time of diagnosis of wild-type transthyretin cardiac amyloidosis.

	ATTRwt without significant AS N=134	ATTRwt with significant AS N=20	p-value
Age, years; mean (SD)	81 ±7	84 ±7	0.07
Male, n (%)	114 (85)	17 (85)	0.99
Hypertension, n (%)	111 (83)	15 (75)	0.40
Dyslipidaemia,, n (%)	67 (50)	11 (55)	0.68
Diabetes, n (%)	30 (22)	4 (20)	0.81
CKD, n (%)	25 (18)	3 (15)	0.69
Coronary artery disease, n (%)	24 (18)	5 (25)	0.45
Previous HF, n (%)	88 (66)	14 (70)	0.71
Previous AF, n (%)	84 (65)	14 (70)	0.70
NT proBNP, pg/mL: median [IQR]	2838 [1317-5332]	4161 [2229-7625]	0.12
Troponin, pg/mL median [IQR]	54 [36-88]	55 [41-72]	0.87
LVEF, %; mean (SD)	53 ±10	56 ±11	0.23
Significant MR, n (%)	27 (20)	3 (15)	0.57
Significant TR, n (%)	35 (26)	6 (30)	0.74
TAPSE, mm; mean (SD)	19 ±5	18 ±3	0.44
PSAP, mmHg; mean (SD)	42 ±12	46 ±12	0.15

ATTRwt; wild-type transthyretin cardiac amyloidosis. AS: aortic stenosis. CKD: chronic kidney disease. HF: heart failure. AF: atrial fibrillation. LVEF: left ventricular ejection fraction. MR: mitral regurgitation. TR: tricuspid regurgitation. TAPSE: tricuspid annular plane systolic excursion. PASP: pulmonary artery systolic pressure.

Patients with significant MR had a higher prevalence of chronic kidney disease (37% vs. 14%; p=0.004) and lower LVEF (47% vs. 54%; p=0.002) compared with those without MR. They also showed a higher prevalence of concomitant significant TR (57% vs. 20%; p<0.001), lower TAPSE (18 vs. 20 mm; p=0.04), and higher pulmonary systolic pressure (50 vs. 40 mmHg; p<0.001) compared with patients without MR **(**Table 2**)**.

**Table 2:** Characteristics of patients with and without significant mitral regurgitation at the time of diagnosis of wild-type transthyretin cardiac amyloidosis

	ATTRwt without significant MR N=122	ATTRwt with significant MR N=30	p-value
Age, years; mean (SD)	81 ±7	82 ±8	0.45
Male, n (%)	109 (89)	20 (67)	0.002
Hypertension, n (%)	99 (81)	26 (86)	0.50
Dyslipidaemia,, n (%)	58 (47)	19 (63)	0.12
Diabetes, n (%)	26 (21)	7 (23)	0.81
CKD, n (%)	17 (14)	11 (37)	0.004
Coronary artery disease, n (%)	22 (18)	7 (23)	0.51
Previous HF, n (%)	78 (64)	23 (77)	0.19
Previous AF, n (%)	63 (52)	21 (70)	0.07
NT proBNP, pg/mL: median [IQR]	2759 [1225-5778]	3905 [2262-5484]	0.18
Troponin, pg/mL median [IQR]	53 [36-87]	59 [43-88]	0.32
LVEF, %; mean (SD)	54 ±10	47 ±12	0.002
Significant AS, n (%)	17 (14)	3 (10)	0.57
Significant TR, n (%)	24 (20)	17 (57)	<0.001
TAPSE, mm; mean (SD)	20 ±4	18 ±4	0.04
PSAP, mmHg; mean (SD)	40 ±12	50 ±12	<0.001

ATTRwt: wild-type transthyretin cardiac amyloidosis. MR: mitral regurgitation. CKD: chronic kidney disease. HF: heart failure. AF: atrial fibrillation. LVEF: left ventricular ejection fraction. AS: aortic stenosis. TR: tricuspid regurgitation. TAPSE: tricuspid annular plane systolic excursion. PASP: pulmonary artery systolic pressure.

Patients with significant TR were older (83 vs. 80 years; p=0.020), had a higher prevalence of atrial fibrillation (78% vs. 47%; p=0.001), and prior HF (83% vs. 60%; p=0.009) compared with those without TR. They also had higher NT-proBNP levels (4442 vs. 2744 pg/mL; p=0.020), lower LVEF (47% vs. 55%; p<0.001), and a higher prevalence of concomitant significant MR (41% vs. 11%; p<0.001). Additionally, they had lower TAPSE (18 vs. 20 mm; p=0.020) and higher pulmonary systolic pressure (49 vs. 39 mmHg; p<0.001) **(**Table 3**)**.

**Table 3:** Characteristics of patients with and without significant tricuspid regurgitation at the time of diagnosis of wild-type transthyretin cardiac amyloidosis

	ATTRwt without significant TR N=111	ATTRwt with significant TR N=41	p-value
Age, years; mean (SD)	80 ±7	83 ±7	0.02
Male, n (%)	94 (85)	35 (85)	0.92
Hypertension, n (%)	92 (83)	33 (81)	0.73
Dyslipidaemia,, n (%)	48 (43)	29 (70)	0.003
Diabetes, n (%)	19 (17)	14 (34)	0.02
CKD, n (%)	17 (15)	11 (27)	0.10
Coronary artery disease, n (%)	19 (17)	10 (24)	0.31
Previous HF, n (%)	67 (60)	34 (83)	0.009
Previous AF, n (%)	52 (47)	32 (78)	0.001
NT proBNP, pg/mL: median [IQR]	2744 [1174-5450]	4442 [2245-7176]	0.02
Troponin, pg/mL median [IQR]	53 [36-82]	66 [43-89]	0.16
LVEF, %; mean (SD)	55 ±10	47 ±11	<0.001
Significant AS, n (%)	14 (12)	6 (14)	0.73
Significant MR, n (%)	13 (11)	17 (41)	<0.001
TAPSE, mm; mean (SD)	20 ±5	18 ±4	0.02
PSAP, mmHg; mean (SD)	39 ±12	49 ±11	<0.001

ATTRwt: wild-type transthyretin cardiac amyloidosis. TR: tricuspid regurgitation. CKD: chronic kidney disease. HF: heart failure. AF: atrial fibrillation. LVEF: left ventricular ejection fraction. AS: aortic stenosis. MR: mitral regurgitation. TAPSE: tricuspid annular plane systolic excursion. PASP: pulmonary artery systolic pressure.

### Incidence of events during follow-up

**Figure 1:**
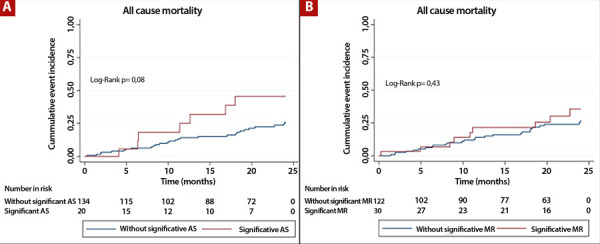
A) Kaplan-Meier survival curves in patients with and without significant aortic stenosis. B) Kaplan-Meier survival curves in patients with and without significant mitral regurgitation. AS: aortic stenosis, MR: mitral regurgitation.

**Figura 2:**
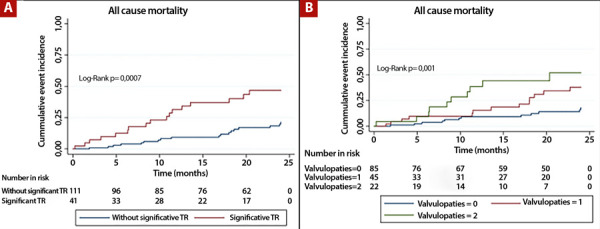
A) Kaplan-Meier survival curves in patients with and without significant tricuspid regurgitation. B) Kaplan-Meier survival curves according to the number of significant valvular diseases (none, one, or two). TR: tricuspideal regurgitation.

Overall, 23% (n=35) of patients died during the 2-year follow-up. In analyses according to the presence or absence of each significant valvular disease, no differences were observed in all-cause mortality between patients with and without significant AS (35% vs. 21%; log-rank p=0.080; Figure 1A), or between those with and without significant MR (30% vs. 21%; log-rank p=0.430; Figure 1B). By contrast, patients with significant TR had a higher incidence of all-cause mortality compared with those without TR (41% vs. 16%; log-rank p=0.0007; Figure 2A). In addition, patients with two concomitant valvular diseases had higher mortality compared with those with one isolated significant valvular disease or none (45% vs. 29% vs. 14%; log-rank p=0.001; Figure 2B).

In multivariable Cox models, the presence of significant TR was independently associated with higher all-cause mortality after adjustment for the presence of other valvular diseases (hazard ratio [HR]: 3.00; 95% confidence interval [CI]: 1.46-6.16; p=0.003). This association remained significant in a second model adjusted for age and NT-proBNP levels (HR: 2.46; 95% CI: 1.22-4.95; p=0.012). The multivariable models are detailed in Table 4. 

**Table 4:** Multivariable Cox models.

	Multivariable analysis		
	HR (95% CI)	p-value
Model 1		
Significant TR	3.00 (1.46 - 6.16)	0.003
Significant MR	0.84 (0.37 - 1.90)	0.68
Significant AS	1.78 (0.77 - 4.09)	0.18
Model 2		
Significant TR	2.46 (1.22 - 4.96)	0.01
Age at diagnosis (continuous)	1.03 (0.97 - 1.08)	0.31
NT-proBNP (continuous, per 1000 pg/mL)	1.07 (1 - 1.14)	0.05

TR: tricuspid regurgitation. MR: mitral regurgitation. AS: aortic stenosis. HR: hazard ratio. CI: confidence interval.

## Discussion

In our cohort of patients with ATTRwt-CM, significant valvular heart disease was frequent, with approximately half presenting at least one moderate or severe valvular lesion. Among them, significant TR was the most prevalent, occurring in about one in four patients. Furthermore, significant TR demonstrated an adverse prognostic impact, as it was independently associated with all-cause mortality during follow-up. This study represents one of the largest cohorts of patients with ATTRwt-CM reported in our region, using prospective registry data from a national referral center.

Regarding specific valvular lesions, although the coexistence of AS and cardiac amyloidosis has been widely described ^9^, there is limited evidence on the prevalence of other valvular diseases in this population. This is of particular interest because, in our cohort, both significant TR and MR were more prevalent than AS at the time of ATTRwt-CM diagnosis.

The prevalence of significant AS in our cohort was 13%, a figure comparable to national and international series analyzing the coexistence of ATTRwt-CM and AS in patients with a primary diagnosis of amyloidosis. Our group had previously reported a prevalence of 10.5% in a smaller cohort ^15^, while Sperry et al. found 15.6% coexistence in a North American retrospective cohort of 171 ATTRwt-CM patients. ^16^ Conversely, a larger number of studies have assessed the coexistence of significant AS and ATTRwt-CM in populations with a primary diagnosis of AS, particularly among patients evaluated for aortic valve replacement, especially transcatheter procedures. ^10^^,^^17^^-^^19^^)^ In this setting, the reported prevalence also ranged between 10% and 20%, underscoring the importance of considering an ATTRwt-CM diagnosis, particularly in patients with clinical “red flags” such as relatively low-voltage electrocardiograms (ECGs) or bilateral carpal tunnel syndrome. Prognostically, a meta-analysis including 21 studies and 4243 patients showed higher mortality in those with both conditions compared with patients with isolated amyloidosis or AS. ^20^ In our cohort, we observed a trend toward worse outcomes in this group, though statistical significance was not reached, likely due to the small number of patients with significant AS and the short follow-up period.

With respect to significant MR, very few studies have described its prevalence in patients with cardiac amyloidosis. A study from the National Amyloidosis Centre in London reported a prevalence of 30% ^21^, similar to our cohort (close to 20%), while a multicenter Italian cohort study reported a higher prevalence of nearly 45% in ATTRwt-CM patients. ^22^ In that study, significant MR was not independently associated with mortality or HF hospitalization after adjusting for biomarkers such as NT-proBNP and troponin, whereas significant TR retained prognostic value. In our cohort, although patients with significant MR had lower LVEF than those without, we did not observe an association with worse clinical outcomes during follow-up. This may be explained, at least in part, by the fact that LVEF is not a robust prognostic marker in ATTRwt-CM. Indeed, major prognostic staging systems in cardiac amyloidosis exclude LVEF, instead prioritizing biomarkers such as NT-proBNP, troponin, and renal function. ^7^^,^^8^

By contrast, significant TR was the most prevalent valvular lesion in our cohort and the only one with independent prognostic value during follow-up. Previous studies have also highlighted its prognostic impact in cardiac amyloidosis. In 2021, a French retrospective study including 283 patients with transthyretin and light-chain amyloidosis assessed the prognostic role of significant TR. ^23^ Remarkably, the prevalence of significant TR in that cohort (28%) was virtually identical to that observed in our study (27%), and the clinical and echocardiographic characteristics of patients were comparable. The authors showed that TR had an independent prognostic impact in ATTRwt-CM but not in light-chain amyloidosis. These findings suggest that significant TR may represent a marker of more advanced disease in ATTRwt-CM and, in the future, could potentially be integrated into prognostic stratification models. Furthermore, TR could emerge as a potential therapeutic target in this population, especially in light of recent advances in percutaneous techniques. ^24^^-^^26^

This study has several limitations. First, it was a retrospective analysis, with the inherent biases of this design. Additionally, valvular diseases with different pathophysiological mechanisms (one stenotic and two regurgitant) were grouped together, as these are the three most common lesions, while the prevalence of other valvular conditions was negligible. Quantitative measures of valvular disease were not included due to the retrospective design and lack of such data in most patients with regurgitant lesions. Finally, the relatively small number of events limited the statistical power of multivariable analyses, precluding the addition of other established prognostic variables to the models.

In conclusion, significant TR was the most prevalent valvular lesion in patients with ATTRwt-CM and was independently associated with reduced survival in this population.
